# Adjuvant Chemotherapy for High-Risk Stage II Colon Cancer: A Population-Based Study

**DOI:** 10.1007/s12029-025-01186-z

**Published:** 2025-02-13

**Authors:** Annmarie Butare, Tia Sutton, Elizabeth Kantzler, Katie N. Kennedy, Dmitry Tumin, Michael D. Honaker

**Affiliations:** 1 Department of Surgery, Brody School of Medicine at East, Carolina University, Greenville, NC USA; 2 Department of Hematology and Oncology, Brody School of Medicine at East Carolina University, Greenville, NC USA; 3 Department of Academic Affairs, East Carolina University Brody School of Medicine, Greenville, NC USA; 4 Department of Surgery, Division of Surgical Oncology, Division of Surgical Oncology, East Carolina University, 600 Moye Blvd, Greenville, NC 27834 USA

**Keywords:** Adjuvant chemotherapy, Stage II colon cancer, High risk

## Abstract

**Background:**

Adjuvant chemotherapy is recommended as an option for patients who have high-risk features. It remains unclear whether all patients with high-risk stage II colon cancer benefit from adjuvant therapy. The primary aim of this study is to evaluate the association between adjuvant chemotherapy and overall survival in patients with high-risk stage II colon cancer.

**Methods:**

Utilizing the Surveillance, Epidemiology and End Results (SEER) database from 2010 to 2019, adult patients with high-risk stage II colon cancer defined as T4 tumor classification, perineural invasion, less than 12 lymph nodes harvested, and poorly differentiated histology. 1:1 ratio propensity matching was used to adjust for confounding variables. Survival differences based on receipt of adjuvant systemic therapy were summarized using a log rank test. Cox proportion hazard regression was used to evaluate overall survival.

**Results:**

Of the 11,619 patients who met inclusion criteria, 2775 (24%) received adjuvant chemotherapy. Patients were more likely to receive adjuvant therapy if they were younger, married or partnered, or had left-sided lesions. Kaplan–Meier estimates showed an improvement in overall survival (log-rank test < 0.001). On pair-stratified Cox proportional hazards regression, adjuvant chemotherapy receipt was associated with 30% lower mortality hazard (hazard ratio [HR] 0.70; 95% CI 0.62, 0.80; *p* < 0.001). However, on landmark analysis, after excluding patients surviving < 3 months, adjuvant chemotherapy was no longer associated with mortality hazard (HR 0.90; 95% CI 0.79, 1.04; *p* = 0.144).

**Conclusion:**

The findings from this large SEER database study provide support for not undergoing adjuvant chemotherapy to patients with high-risk stage II colon cancer.

## Introduction

Colorectal cancer is the fourth most common cause of cancer in the USA. According to the National Cancer Institute, the estimated number of new cases in the USA is 152,810, comprising 7.6% of all cancer diagnoses. Additionally, it is estimated that colorectal cancer will account for 8.7% of all cancer related mortality in the USA by the end of 2024 [1].

The majority of new colon cancer cases are diagnosed at a local or regional stage [2]. Between 2017 and 2021, 38.7% of all new colon cancer cases in the USA were stage II [3]. Of those who are diagnosed with stage II colon cancer, approximately 20–30% are considered high risk [4, 5]. High-risk stage II colon cancer are tumors that on pathologic examination are T4, have lymphovascular invasion (LVI), perineural invasion (PNI), inadequate lymph node harvests (< 12), poor differentiation (grade 3), high tumor budding scores, positive margins, or clinically are obstructing or perforated [2, 6, 7].

While 75–80% of stage II colon cancer will not recur following surgical resection, debate exists on the benefit of adjuvant systemic therapy in patients with stage II disease. This stems from a lack of studies evaluating adjuvant chemotherapy for high-risk stage II disease, as the current data are derived from studies addressing therapy for stage II and stage III colon cancer combined [8, 9]. In addition to surgical resection, the 2024 National Comprehensive Cancer Network (NCCN) guidelines recommend adjuvant therapy or observation as treatment options for patients with high-risk stage II colon cancer [10]. Additionally, current American Society of Clinical Oncology (ASCO) recommendations for adjuvant treatment of stage II disease include consideration of systemic therapy for those with high-risk features [2].

Various high-risk features of stage II colon cancer have been found to worsen survival, and currently serve as a potential indication for adjuvant chemotherapy (ACT); however, conflicting results are seen with the addition of adjuvant therapy and improvements in overall survival, where some studies report improved survival and others have shown no change [11, 12]. The study’s primary aim was to evaluate the association of adjuvant chemotherapy with overall survival in patients with high-risk stage II colon cancer.

## Methods

### Data Source

The Surveillance, Epidemiology, and End Results (SEER) program, sponsored by the National Cancer Institute (NCI), is the largest publicly available cancer database in the world [13]. It provides deidentified, detailed disease course data spanning 48% of the US population [14]. A retrospective, observational study was conducted utilizing the SEER database from 2010 to 2019 [15]. This study was considered not human subjects research by the East Carolina University and ECU Medical Center IRB due to its use of public, de-identified data.

### Patient Selection

**Fig. 1:**
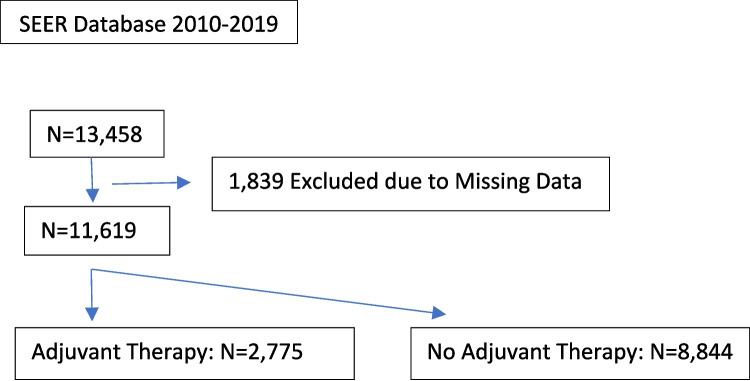
Patient inclusion

Patients ≥ 18 years old diagnosed with stage II colon cancer from 2010 to 2019 based on the World Health Organization (WHO) International Classification of Disease for Oncology third edition (ICD-O-3), site recode for colon, were identified [16]. Histological subtypes were selected using topography codes 8140–8389 if they had positive histology for diagnostic confirmation. Stage II cases were selected based on the derived stage groupings appropriate for the specified time frame (Derived Extent of Disease for 2018–2019, Derived SEER for 2016–2017, and Derived AJCC 7th Edition for 2010–2015). Only cases of the first malignant primary indicator that underwent surgery of the primary site were selected. Cases with pathologies other than adenocarcinoma, primary appendiceal involvement, cases without a known time to treatment, or cases where systemic therapy was given prior to surgery were excluded. High-risk features were chosen according to the National Comprehensive Cancer Network (NCCN) definition of high-risk features for stage II colon cancer [17]. Patients were considered high risk if 1 or more of the following features were present: T4 on TNM classification, perineural invasion (PNI), inadequate lymph node sampling (< 12 lymph nodes), or poorly differentiated/anaplastic histology. Patients with none of these features and with missing data on study variables were excluded from the analysis (Fig. 1) Results were reported per the Strengthening the Reporting of Observational Studies in Epidemiology (STROBE) guidelines [17].

### Variables and Outcome

Our primary outcome was overall survival by receipt of adjuvant chemotherapy, measured as months between diagnosis and death or at the end of follow in which that point in time became a censoring event. Receipt of chemotherapy and sequence of systemic therapy in relation to surgery were used to define patients who received adjuvant treatment. Covariates included age at diagnosis; sex; race and ethnicity (Non-Hispanic White, Non-Hispanic Black, Hispanic, other); marital status at diagnosis (married/partnered, single, formerly married); residing in a metropolitan vs. nonmetropolitan area, based on the US Census Bureau definitions; tumor site (right colon, transverse colon, left colon); pathologic tumor grade; perineural invasion; T4 TNM classification; number of lymph nodes examined; and year of diagnosis [18].

### Statistical Analyses

Continuous variables were presented as median and interquartile range (IQR). Categorical variables were presented as counts with percentages. Study variables were compared between groups using rank-sum tests, chi-square tests, or Fisher’s exact tests, as appropriate. Prior to matching, overall survival was compared based on receipt of adjuvant chemotherapy using Kaplan–Meier curves and the log-rank test. We then fit a propensity score model for receipt of adjuvant chemotherapy, using a logistic regression on all observed covariates with the exception of diagnosis year. Cases receiving adjuvant chemotherapy were matched without replacement at a 1:1 ratio to controls, based on the logit of the propensity score. A caliper equaling 0.2 standard deviations of the propensity score logit in width was used to ensure sufficient similarity between cases and controls. Furthermore, we required all cases to be matched to controls from the same diagnosis year. Covariate balance in the matched sample was assessed using means, proportions, and standardized differences (StdDiff), where StdDiff < 0.10 indicated adequate balance. Cox proportional hazards regression, stratified on the matched pairs, was used to evaluate overall survival in the matched sample. Secondary to findings on the initial survival analysis, a landmark survival analysis using the same matching process and Cox regression analysis were repeated for the subsample of patients surviving > 3 months. Analyses were conducted using the Stata/SE 18.0 (College Station, TX: Statcorp, LP). A *p*-value < 0.05 was deemed statistically significant.

## Results

### Demographic and Clinical Characteristics

We identified 13,458 stage II colon cancer patients with at least one high-risk feature and excluded 1839 cases with missing data on study covariates (Fig. 1). Among the remaining 11,619 patients, 2775 patients (24%) received adjuvant chemotherapy, while 8844 patients (76%) did not receive adjuvant therapy. The median age was 71 years old, and 47% were male. Thirty-three percent of the patients died during follow-up. Median follow-up time was 39 months (IQR 15,70). The patients receiving chemotherapy were younger (61 years vs 75 years), more likely to be NHB, married/partnered, and have tumors located on the left side (Table 1).

**Table 1:** Demographic and clinical characteristics of patients with high-risk stage II colon cancer

Variable	Total (*N* = 11,619)	No adjuvant therapy (*N* = 8844)	Adjuvant therapy (*N* = 2775)	*p*-value
	Median (IQR) or count (%)	Median (IQR) or count (%)	Median (IQR) or count (%)	
Age	71 (60, 81)	75 (64, 83)	61 (52, 69)	< 0.001
Sex				< 0.001
Male	5458 (47)	4032 (46)	1426 (51)	
Female	6161 (53)	4812 (54)	1349 (49)	
Race and ethnicity				< 0.001
NHW	8008 (69)	6249 (71)	1759 (63)	
NHB	1160 (10)	818 (9)	342 (12)	
Hispanic	1420 (12)	1036 (12)	384 (14)	
Other	1031 (9)	741 (8)	290 (10)	
Marital status				< 0.001
Married/partnered	5953 (51)	4330 (49)	1623 (58)	
Single	2001 (17)	1404 (16)	597 (22)	
Formerly married	3665 (32)	3110 (35)	555 (20)	
Geographic area				0.986
Metropolitan	10010 (86)	7619 (86)	2391 (86)	
Nonmetropolitan	1609 (14)	1225 (14)	384 (14)	
Site^a^				< 0.001
Right colon	5964 (51)	4800 (54)	1164 (42)	
Transverse colon	1526 (13)	1179 (13)	347 (13)	
Left colon	4129 (36)	2865 (32)	1264 (46)	
Tumor grade				< 0.001
Low	501 (4)	391 (4)	110 (4)	
Moderate	5955 (51)	4373 (49)	1582 (57)	
Poor/anaplastic	5163 (44)	4080 (46)	1083 (39)	
PNI^b^				< 0.001
Not present	9166 (79)	7087 (80)	2079 (75)	
Present	2453 (21)	1757 (20)	696 (25)	
Lymph nodes				< 0.001
$\ge$ 12	8688 (75)	6432 (73)	2256 (81)	
< 12	2931 (25)	2412 (27)	519 (19)	
Year of diagnosis	2014 (2012, 2017)	2014 (2012, 2017)	2015 (2012, 2017)	< 0.001

^a^Right colon includes cecum, ascending colon, and hepatic flexure; left colon includes splenic flexure, descending colon, and sigmoid colon

^b^Perineural invasion

### Survival Analysis

**Fig. 2:**
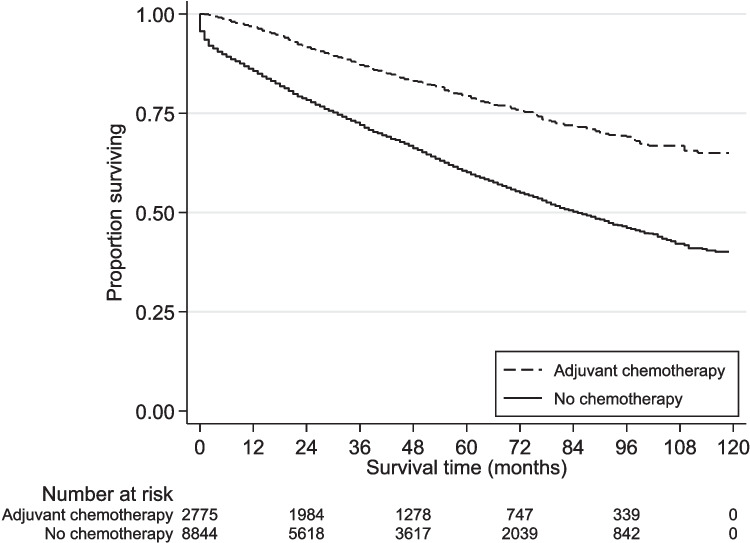
Kaplan–Meier curve of overall survival, stratified by use of adjuvant therapy (log rank test *p* < 0.001)

Kaplan–Meier analysis demonstrated significantly better survival among patients treated with adjuvant chemotherapy (Fig. 2; log-rank test *p* < 0.001). After propensity score matching, we excluded 183 cases (patients receiving adjuvant chemotherapy) with no suitable control, and 6252 controls not selected during the matching process. In the remaining sample of 2592 matched pairs, all covariates achieved satisfactory balance between cases and controls (Table 2), and thus, none had to be entered into the Cox regression on the matched sample. On pair-stratified Cox proportional hazards regression, adjuvant chemotherapy receipt was associated with 30% lower mortality hazard (hazard ratio [HR] 0.70; 95% CI 0.62, 0.80; *p* < 0.001).

**Table 2:** Covariate balance after propensity score matching on receipt of adjuvant chemotherapy (*N* = 2592 matched pairs)

Variable	Cases (adjuvant therapy)	Controls (no adjuvant therapy)	Standardized difference
	Mean (SD) or proportion	Mean (SD) or proportion	
Age	61.6 (11.3)	61.3 (12.2)	0.03
Sex			
Male	0.51	0.54	0.05
Female	0.49	0.46	0.05
Race and ethnicity			
NHW	0.64	0.65	0.02
NHB	0.12	0.12	0.00
Hispanic	0.14	0.14	0.00
Other	0.10	0.09	0.03
Marital status			
Married/partnered	0.58	0.60	0.05
Single	0.21	0.20	0.03
Formerly married	0.21	0.20	0.04
Geographic area			
Metropolitan	0.86	0.87	0.03
Nonmetropolitan	0.14	0.13	0.03
Site^a^			
Right colon	0.43	0.42	0.02
Transverse colon	0.12	0.13	0.01
Left colon	0.44	0.45	0.01
Tumor grade			
Low	0.04	0.04	0.00
Moderate	0.56	0.56	0.01
Poor/anaplastic	0.40	0.40	0.01
PNI^b^			
Not present	0.75	0.75	0.00
Present	0.25	0.25	0.00
Lymph nodes			
$\ge$12	0.80	0.80	0.00
< 12	0.20	0.20	0.00
Year of diagnosis	2015 (3)	2015 (3)	0.00

^a^Right colon includes cecum, ascending colon, and hepatic flexure; left colon includes splenic flexure, descending colon, and sigmoid colon

^b^Perineural invasion

In the landmark analysis, we restricted the sample to 10,399 patients surviving > 3 months (of whom 2657 received adjuvant chemotherapy). We then re-fit the propensity score model and excluded 197 cases with no suitable control and 5282 controls not used in the matching process. All covariates were successfully balanced by the matching procedure (Table 3). In the propensity-matched Cox model of conditional survival > 3 months, adjuvant chemotherapy was no longer associated with mortality hazard (HR 0.90; 95% CI 0.79, 1.04; *p* = 0.144).

**Table 3:** Covariate balance after propensity score matching on receipt of adjuvant chemotherapy, limiting to patients who survived > 3 months after diagnosis (*N* = 2460 matched pairs)

Variable	Cases (adjuvant therapy)	Controls (no adjuvant therapy)	Standardized difference
	Mean (SD) or proportion	Mean (SD) or proportion	
Age	61.7 (11.4)	61.3 (12.1)	0.04
Sex			
Male	0.51	0.53	0.04
Female	0.49	0.47	0.04
Race and ethnicity			
NHW	0.65	0.65	0.02
NHB	0.12	0.12	0.01
Hispanic	0.13	0.13	0.01
Other	0.10	0.10	0.01
Marital status			
Married/partnered	0.58	0.61	0.06
Single	0.21	0.21	0.01
Formerly married	0.21	0.18	0.08
Geographic area			
Metropolitan	0.86	0.86	0.01
Nonmetropolitan	0.14	0.14	0.01
Site^a^			
Right colon	0.44	0.44	0.00
Transverse colon	0.12	0.12	0.02
Left colon	0.44	0.45	0.01
Tumor grade			
Low	0.04	0.04	0.00
Moderate	0.56	0.55	0.03
Poor/anaplastic	0.40	0.41	0.03
PNI^b^			
Not present	0.76	0.76	0.00
Present	0.24	0.24	0.00
Lymph nodes			
$\ge$12	0.80	0.80	0.00
< 12	0.20	0.20	0.00
Year of diagnosis	2014 (3)	2014 (3)	0.00

^a^Right colon includes cecum, ascending colon, and hepatic flexure; left colon includes splenic flexure, descending colon, and sigmoid colon

^b^Perineural invasion

## Discussion

In this large database analysis including over 11,000 patients, although adjuvant chemotherapy was initially found to provide a significant survival benefit in patients with high-risk stage II colon cancer, when the first 3 months were excluded, a survival benefit was no longer seen. This difference is unlikely to be associated with the receipt of adjuvant therapy and more associated with surgical outcomes as it is impractical that a patient would be diagnosed with colon adenocarcinoma, and within 3 months from diagnosis, adjuvant therapy has an impact on their overall survival.

Our results are in contrast with results of other studies evaluating survival of high-risk stage II colon cancer treated with ACT. A large retrospective study using data from the Danish Colorectal Cancer Group, the National Patient Registry, and the Danish Pathology Registry found an 84.9% overall survival in patients receiving ACT, compared with 66.3% of patients who did not [19]. Similar to our study, the aforementioned study only included patients who survived more than 3 months; however, the defining features of high-risk colon cancer are not consistent, which may explain the conflicting results. For example, our study used NCCN and ASCO guidelines to determine high-risk features (T4, PNI, < 12 lymph nodes harvested, poorly differentiated histology); however, the study described above used the following criteria to define high-risk features: T4, Grade B or C anastomotic leak, use of stent or loop ileostomy for emergent diversion, signet ring pathology, and < 12 lymph nodes harvested [13]. Another large-scale study using the SEER database quantified the survival benefit of chemotherapy use in high-risk stage II patients, finding its use beneficial. However, this study did not exclude patients whose survival may have been impacted by the surgery itself which could confound the findings as was found in the current study, which may have contributed to an improved survival profile [20].

Current recommendations for ACT in patients with high-risk stage II colon cancer are partially extrapolated from studies including patients with stage III disease [21, 22]. In the QUASAR study, adjuvant chemotherapy provided a modest benefit in patients with stage II colorectal cancer, although patients were not stratified by risk [22]. Notably, in this trial, nearly two-thirds of patients had less than 12 lymph nodes resected, which could imply the presence of a higher stage of disease than recognized at trial enrollment. The MOSAIC trial evaluated the benefit of adding oxaliplatin to the treatment of patients with stage II and III colon cancer. This study failed to show a survival benefit to patients with stage II disease [21]. Other trials including the IMPACT B2 Analysis, the Intergroup Analysis, the Cancer Care Ontario Program, and NSABP, and the SACURA trial also failed to show survival benefit with the addition of ACT [9]. With conflicting results and a lack of randomized data, our study results add to the current literature in providing insight to the risk and benefit discussion regarding adjuvant chemotherapy for high-risk stage II colon cancer as administration of chemotherapy is not without risks of morbidity and reduced quality of life. With side-effects ranging from nausea and vomiting to peripheral neuropathy and skeletal muscle wasting (PMID: 9704726, PMID: 18798075).

The current study has several limitations. First, the study design was a retrospective study, inherently at risk for biases. For example, the median age differed significantly between groups, due to adjuvant therapy not being offered as frequently to older patients as to younger patients. This limitation was addressed through propensity score matching. Second, large database analyses are limited to data collected and entered by registrars. This is likely to introduce bias and potential reporting errors in the data [23, 24]. Third, there are high-risk features not included in the SEER database such as colonic perforation and obstruction. Additionally, other high-risk features such as tumor budding, lymphovascular invasion, perforation, and obstruction are not captured by the database. Therefore, we can only make inferences assuming that these factors are otherwise not present or not systematically different between study groups. Lastly, the specific adjuvant therapy regimen and duration were unknown.

## Conclusion

While consideration of adjuvant chemotherapy for patients with high-risk stage II colon cancer is multifactorial, this study supports the consideration for withholding adjuvant therapy due to the finding of a lack of a survival benefit. However, further research is needed to distinguish high-risk features that are clinically relevant for improving overall survival in this subset of patients.

## Data Availability

The Surveillance, Epidemiology and End Results database does not allow the sharing of data.
